# Supplementary material for: Lipid scrambling is a general feature of protein insertases
Source: bioRxiv. 2023 Sep 1:2023.09.01.555937. Preprint. [Version 1] doi: 10.1101/2023.09.01.555937 (PMC10491306; doi:10.1101/2023.09.01.555937)
Supplement: Supplement 1 [file NIHPP2023.09.01.555937v1-supplement-1.pdf]

bioRxiv preprint doi: https://doi.org/10.1101/2023.09.01.555937; this version posted September 1, 2023. The copyright holder for this preprint (which was not certified by peer review) is the author/funder, who has granted bioRxiv a license to display the preprint in perpetuity. It is made available under aCC-BY 4.0 International license.

Supporting Material

Table S1. Details about all simulated systems. Systems for which the uniprot ID is provided means that the available AlphaFold (AF) model was used. All oligomers were predicted with AF multimer.

ID	System (number of chains)	Organism	Source (Uniprot ID, PDB code or AlphaFold)	Total # of DOPC lipids	Total events	MD run length
1	TMEM16K (2)	*H. sapiens*	PDB: 5OC9	1000	251	20 µs
2	TMEM16K (2)	*H. sapiens*	PDB: 6R65	1000	278	20 µs
3	TMEM16K (2)	*H. sapiens*	PDB: 6R7X	1000	10	20 µs
4	TMEM16K (2)	*H. sapiens*	PDB: 6R7Y	1000	7	20 µs
5	TMEM16K (2)	*H. sapiens*	PDB: 6R7Z	1000	28	20 µs
6	TMEM16F (2)	*M. musculus*	PDB: 8B8Q	800	27	20 µs
7	TMEM16A (2)	*M. musculus*	PDB: 7ZK3	800	29	20 µs
8	TMEM41B (1)	*H. sapiens*	ID: Q5BJD5	600	22	20 µs
9	VMP1 (1)	*H. sapiens*	ID: Q96GC9	600	24	20 µs
10	TMEM41B - VMP1 (2)	*H. sapiens*	ID: Q5BJD5<br>ID: Q96GC9	800	31	20 µs
11	ATG9 (3)	*S. pombe*	PDB: 7D0I	1200	175	20 µs
12	ATG9A (3)	*H. sapiens*	PDB: 6WQZ	1200	93	20 µs
13	VDAC1 (1)	*H. sapiens*	PDB: 6G6U	600	24	20 µs
14	VDAC1 (2)	*H. sapiens*	PDB: 6G6U	800	161	20 µs
15	VDAC2 (1)	*D. rerio*	PDB: 4BUM	800	25	20 µs
16	VDAC2 (2)	*D. rerio*	PDB: 4BUM	800	231	20 µs
17	VDAC3 (1)	*H. sapiens*	ID: Q9Y277	800	24	20 µs
18	VDAC3 (2)	*H. sapiens*	ID: Q9Y277	800	319	20 µs
19	Rhodopsin (1)	*B. taurus*	PDB: 4A4M	800	19	20 µs
20	Rhodopsin (2)	*B. taurus*	PDB: 4A4M	800	39	20 µs
21	MCP1 (1)	*S. cerevisiae*	ID: Q12106	600	26	20 µs
22	MCP1 (2)	*S. cerevisiae*	ID: Q12106	800	33	20 µs
23	Xk (1)	*H. sapiens*	ID: P51811	800	9	20 µs
24	Xkr8 (1)	*H. sapiens*	PDB: 7DCE	800	11	20 µs
25	Xkr9 (1)	*R. norvegicus*	PDB: 7P16	800	8	20 µs
26	GlpG (1)	*E. colii*	PDB: 3B45	600	3	20 µs

bioRxiv preprint doi: https://doi.org/10.1101/2023.09.01.555937; this version posted September 1, 2023. The copyright holder for this preprint (which was not certified by peer review) is the author/funder, who has granted bioRxiv a license to display the preprint in perpetuity. It is made available under aCC-BY 4.0 International license.

27	Spns1 (1)	*H. sapiens*	ID: Q9H2V7	800	3	20 μs
28	MFSD2A (1)	*H. sapiens*	PDB: 7OIX	800	5	20 μs
29	ABCB1 (1)	*H. sapiens*	PDB: 7A69	1000	4	20 μs
30	ABCB4 (4)	*H. sapiens*	PDB: 6S7P	800	9	20 μs
31	ABCB11 (11)	*H. sapiens*	PDB: 6LR0	800	8	20 μs
32	ABCG40 (1)	*A. thaliana*	ID: Q9M9E1	1200	2	20 μs
33	MsbA (2)	*E. colii*	PDB: 7PH4	800	1	20 μs
34	PglK (2)	*C. jejuni*	PDB: 5C73	800	15	20 μs
35	PglK (2)	*C. jejuni*	PDB: 5C76	1000	18	20 μs
36	PglK (2)	*C. jejuni*	PDB: 5C78	1000	19	20 μs
37	SERCA2b (1)	*H. sapiens*	PDB: 7E7S	800	9	20 μs
38	DGGGP (1)	*M. jannaschii*	PDB: 6M31	800	1	20 μs
39	DGGGP (2)	*M. jannaschii*	PDB: 6M31	800	1	20 μs
40	Drs2p (1)	*S. cerevisiae*	PDB: 6ROH	1200	8	20 μs
41	Drs2p (1)	*S. cerevisiae*	PDB: 6ROI	1200	7	20 μs
42	Drs2p (1)	*S. cerevisiae*	PDB: 6ROJ	1000	3	20 μs
43	VAMP2 (1)	*H. sapiens*	ID: P63027	600	0	20 μs
44	VAMP2 (2)	*H. sapiens*	ID: P63027	1000	2	20 μs
45	YidC (1)	*B. halodurans*	PDB: 3WO6	600	16	20 μs
46	YidC R72A (1)	*B. halodurans*	PDB: 3WO6	800	8	20 μs
47	YidC (1)	*B. halodurans*	PDB: 3WO7	800	10	20 μs
48	MisCB (1)	*B. subtilis*	ID: P54544	800	33	20 μs
49	Oxa1 (1)	*S. cerevisiae*	ID: P39952	800	45	20 μs
50	OXA1L (1)	*H. sapiens*	PDB: 6ZM5	800	18	20 μs
51	OXA1L-peptide (2)	*H. sapiens*	PDB: 6ZM5	800	0	20 μs
52	Cox18 (1)	*S. cerevisiae*	ID: P53239	800	47	20 μs
53	Alb3 (1)	*A. thaliana*	ID: Q8LBP4	800	24	20 μs
54	Emc1 (1)	*S. cerevisiae*	PDB: 6WB9	800	0	20 μs
55	Emc3 (1)	*S. cerevisiae*	PDB: 6WB9	800	113	20 μs
56	Emc4 (1)	*S. cerevisiae*	PDB: 6WB9	800	115	20 μs
57	Emc5 (1)	*S. cerevisiae*	PDB: 6WB9	800	0	20 μs
58	Emc6 (1)	*S. cerevisiae*	PDB: 6WB9	800	1	20 μs

bioRxiv preprint doi: https://doi.org/10.1101/2023.09.01.555937; this version posted September 1, 2023. The copyright holder for this preprint (which was not certified by peer review) is the author/funder, who has granted bioRxiv a license to display the preprint in perpetuity. It is made available under aCC-BY 4.0 International license.

59	EMC complex (8)	*S. cerevisiae*	PDB: 6WB9	1000	170	20 µs
60	Get1 (1)	*S. cerevisiae*	ID: P53192	800	56	20 µs
61	Get1 (1)<br>F113L W147L W189L	*S. cerevisiae*	ID: P53192	600	85	20 µs
62	Get1 (1)<br>F113L G120L W147L G185L W189L	*S. cerevisiae*	ID: P53192	600	22	20 µs
63	Get1 (1)<br>K16L Q19L R104L K116L W189L N193L N196L Q199L	*S. cerevisiae*	ID: P53192	800	36	20 µs
64	Get1 (1)<br>T109L K116L K121L W147L Y149L S183L G185L W189L N193L N196L	*S. cerevisiae*	ID: P53192	800	0	20 µs
65	Get2 (1)	*S. cerevisiae*	ID: P40056	800	7	20 µs
66	Get1 - Get2 (2)	*S. cerevisiae*	ID: P53192<br>ID: P40056	800	71	20 µs
67	GET complex (4)	*S. cerevisiae*	ID: P53192<br>ID: P40056	800	142	20 µs
68	GET complex (4)<br>R148L K328L R416L K428 K433L E511L	*S. cerevisiae*	ID: P53192<br>ID: P40056	800	176	20 µs
69	GET complex (4)<br>T421L K428L K433L W459L Y461L S495L G497L W501L N505L N508L	*S. cerevisiae*	ID: P53192<br>ID: P40056	800	22	20 µs
70	GET complex (4)<br>T421L K428L G432L K433L G458L W459L Y461L S495L G497L W501L N505L N508L	*S. cerevisiae*	ID: P53192<br>ID: P40056	800	22	20 µs
71	WRB (1)	*H. sapiens*	PDB: 6SO5	800	7	20 µs
72	CAML (1)	*H. sapiens*	PDB: 6SO5	800	2	20 µs
73	WRB - CAML (2)	*H. sapiens*	PDB: 6SO5	800	22	20 µs
74	WRB - CAML (4)	*H. sapiens*	PDB: 6SO5	800	55	20 µs
75	TRC complex (6)	*H. sapiens*	PDB: 6SO5	800	39	20 µs
76	TMCO1 (1)	*H. sapiens*	PDB: 6W6L	600	70	20 µs
77	C20orf24 (1)	*H. sapiens*	PDB: 6W6L	600	14	20 µs
78	GEL complex (2)	*H. sapiens*	PDB: 6W6L	800	80	20 µs
79	Nicalin (1)	*H. sapiens*	PDB: 6W6L	600	0	20 µs

bioRxiv preprint doi: https://doi.org/10.1101/2023.09.01.555937; this version posted September 1, 2023. The copyright holder for this preprint (which was not certified by peer review) is the author/funder, who has granted bioRxiv a license to display the preprint in perpetuity. It is made available under aCC-BY 4.0 International license.

80	NOMO (1)	*H. sapiens*	PDB: 6W6L	600	0	20 µs
81	TMEM147 (1)	*H. sapiens*	PDB: 6W6L	600	12	20 µs
82	BOS complex (3)	*H. sapiens*	PDB: 6W6L	800	19	20 µs
83	PAT complex (2)	*H. sapiens*	ID: Q9Y284<br>PDB: 6W6L	800	5	20 µs
84	Atp13a1 (1)	*H. sapiens*	ID: Q9HD20	800	26	20 µs
85	Atp13a1 (1)	*M. musculus*	ID: Q9EPE9	800	20	20 µs
86	Shr3 (1)	*S. cerevisiae*	ID: Q02774	800	3	20 µs
87	Hrd1 (1)	*S. cerevisiae*	PDB: 6VJZ	800	22	20 µs
88	Der1 (1)	*S. cerevisiae*	PDB: 6VJZ	800	1	20 µs
89	Hrd1-Der1 (2)	*S. cerevisiae*	PDB: 6VJZ	800	28	20 µs
90	ERAD complex (4)	*S. cerevisiae*	PDB: 6VJZ	800	32	20 µs
91	Sec61 α - Sec61 γ (2)	*C. familiaris*	PDB: 6Z3T	800	98	20 µs
92	SEC61 complex (3)	*H. sapiens*	PDB: 8DNV	800	26	20 µs
93	SEC61 complex (3)	*H. sapiens*	PDB: 8DNW	800	42	20 µs
94	Sec61 α (1)	*H. sapiens*	PDB: 8B6L	800	46	20 µs
95	Sec61 β (1)	*H. sapiens*	PDB: 8B6L	800	0	20 µs
96	Sec61 γ (1)	*H. sapiens*	PDB: 8B6L	800	0	20 µs
97	SEC61 complex (3)	*H. sapiens*	PDB: 8B6L	800	13	20 µs
98	Trap α (1)	*H. sapiens*	PDB: 8B6L	800	0	20 µs
99	Trap β (1)	*H. sapiens*	PDB: 8B6L	800	0	20 µs
100	Trap γ (1)	*H. sapiens*	PDB: 8B6L	800	20	20 µs
101	Trap δ (1)	*H. sapiens*	PDB: 8B6L	800	0	20 µs
102	Trap β - Trap δ - Trap γ (3)	*H. sapiens*	PDB: 8B6L	800	4	20 µs
103	TRAP complex (4)	*H. sapiens*	PDB: 8B6L	800	7	20 µs
104	SEC61 complex - TRAP complex (7)	*H. sapiens*	PDB: 8B6L	1000	26	20 µs
105	RPN1 (1)	*H. sapiens*	PDB: 8B6L	800	0	20 µs
106	RPN2 (1)	*H. sapiens*	PDB: 8B6L	1200	0	20 µs
107	OST4 (1)	*H. sapiens*	PDB: 8B6L	600	1	20 µs
108	OST48 (1)	*H. sapiens*	PDB: 8B6L	800	1	20 µs
109	DAD1 (1)	*H. sapiens*	PDB: 8B6L	600	32	20 µs
110	STT3a (1)	*H. sapiens*	PDB: 8B6L	800	50	20 µs

bioRxiv preprint doi: https://doi.org/10.1101/2023.09.01.555937; this version posted September 1, 2023. The copyright holder for this preprint (which was not certified by peer review) is the author/funder, who has granted bioRxiv a license to display the preprint in perpetuity. It is made available under aCC-BY 4.0 International license.

111	TMEM258 (1)	*H. sapiens*	PDB: 8B6L	600	3	20 µs
112	OSTC (1)	*H. sapiens*	PDB: 8B6L	800	132	20 µs
113	OSTA complex (8)	*H. sapiens*	PDB: 8B6L	1600	35	20 µs
114	SEC61 complex - OSTA complex (11)	*H. sapiens*	PDB: 8B6L	1600	35	20 µs
115	SEC61 complex - TRAP complex - OSTA complex (15)	*H. sapiens*	PDB: 8B6L	1800	72	20 µs
116	Tom5 (1)	*S. cerevisiae*	PDB: 6UCU	800	0	20 µs
117	Tom6 (1)	*S. cerevisiae*	PDB: 6UCU	800	0	20 µs
118	Tom7 (1)	*S. cerevisiae*	PDB: 6UCU	800	0	20 µs
119	Tom22 (1)	*S. cerevisiae*	PDB: 6UCU	800	0	20 µs
120	Tom40 (1)	*S. cerevisiae*	PDB: 6UCU	800	30	20 µs
121	TOM complex (5)	*S. cerevisiae*	PDB: 6UCU	800	56	20 µs
122	TOM complex (10)	*S. cerevisiae*	PDB: 6UCU	1200	119	20 µs
123	BamA (1)	*E. colii*	PDB: 6QGW	800	30	20 µs
124	TamA (1)	*E. colii*	PDB: 4C00	800	112	20 µs
125	Oep80 (1)	*A. thaliana*	ID: Q9C5J8	800	26	20 µs
126	Sam50 (1)	*S. cerevisiae*	PDB: 7BTW	600	125	20 µs
127	Sam50 (1)	*S. cerevisiae*	PDB: 7BTX	800	84	20 µs
128	Sam50 - Sam50 (2)	*S. cerevisiae*	PDB: 7BTW	800	76	20 µs
129	Sam50 - Mdm10 (2)	*S. cerevisiae*	PDB: 7BTY	800	54	20 µs
130	Sam50 - Tom40 (2)	*S. cerevisiae*	PDB: 7E4H	800	77	20 µs
131	SAM complex (3)	*S. cerevisiae*	PDB: 7BTX	800	54	20 µs
132	Tim22 (1)	*S. cerevisiae*	PDB: 6LO8	800	127	20 µs
133	Tim18 (1)	*S. cerevisiae*	PDB: 6LO8	800	56	20 µs
134	Tim54 (1)	*S. cerevisiae*	PDB: 6LO8	800	5	20 µs
135	Sdh3 (1)	*S. cerevisiae*	PDB: 6LO8	800	15	20 µs
136	TIM22 complex (10)	*S. cerevisiae*	PDB: 6LO8	1000	246	20 µs
137	Tim22 (1)	*H. sapiens*	PDB: 7CGP	800	49	20 µs
138	TIM22 complex (15)	*H. sapiens*	PDB: 7CGP	1200	60	20 µs
139	Tim23 (1)	*S. cerevisiae*	PDB: 8SCX	800	2	20 µs
140	Tim17 (1)	*S. cerevisiae*	PDB: 8SCX	800	250	20 µs
141	Tim17	*S. cerevisiae*	PDB: 8SCX	800	127	20 µs

bioRxiv preprint doi: https://doi.org/10.1101/2023.09.01.555937; this version posted September 1, 2023. The copyright holder for this preprint (which was not certified by peer review) is the author/funder, who has granted bioRxiv a license to display the preprint in perpetuity. It is made available under aCC-BY 4.0 International license.

	D17L, D76L, E126L (1)					
142	Mgr2 (1)	*S. cerevisiae*	ID: Q02889	800	27	20 μs
143	Tim17 - Mgr2 (2)	*S. cerevisiae*	PDB: 8SCX<br>ID: Q02889	800	7	20 μs
144	Tim17 - Mgr2 - Tim23 (3)	*S. cerevisiae*	PDB: 8SCX<br>ID: Q02889	800	29	20 μs
145	Tim17 - Tim23 - Tim44 - Mgr2 (4)	*S. cerevisiae*	PDB: 8SCX<br>ID: Q02889	800	21	20 μs
146	TIM23 complex (3)	*S. cerevisiae*	PDB: 8SCX	1000	157	20 μs
147	Tim17 (1)	*H. sapiens*	ID: Q99595	800	213	20 μs
148	Tim17<br>D16L, D77L, E127L (1)	*H. sapiens*	ID: Q99595	800	124	20 μs
149	Tim23 (1)	*H. sapiens*	ID: O14925	800	30	20 μs
150	Tim17 - Tim23 (2)	*H. sapiens*	ID: Q99595<br>ID: O14925	800	182	20 μs
151	Tim50 (1)	*H. sapiens*	ID: Q3ZCQ8	1200	0	20 μs
152	TIM23 complex (3)	*H. sapiens*	ID: Q99595<br>ID: O14925<br>ID: Q3ZCQ8	1000	170	20 μs
153	MTCH1 (1)	*H. sapiens*	ID: Q9NZJ7	600	129	20 μs
154	MTCH2 (1)	*H. sapiens*	ID: Q9NZJ7	800	126	20 μs
155	MTCH2 Q29L, H142L, D189L, N242L (1)	*H. sapiens*	ID: Q9NZJ7	800	93	20 μs
156	MTCH2-peptide (2)	*H. sapiens*	ID: Q9NZJ7	800	2	20 μs
157	SLC25A46 (1)	*H. sapiens*	ID: Q96AG3	800	22	20 μs
158	SLC26A9 (2)	*H. sapiens*	PDB: 7CH1	1000	35	20 μs
159	SLC25A17 (1)	*H. sapiens*	ID: O43808	800	20	20 μs

bioRxiv preprint doi: https://doi.org/10.1101/2023.09.01.555937; this version posted September 1, 2023. The copyright holder for this preprint (which was not certified by peer review) is the author/funder, who has granted bioRxiv a license to display the preprint in perpetuity. It is made available under aCC-BY 4.0 International license.

Supporting Figures

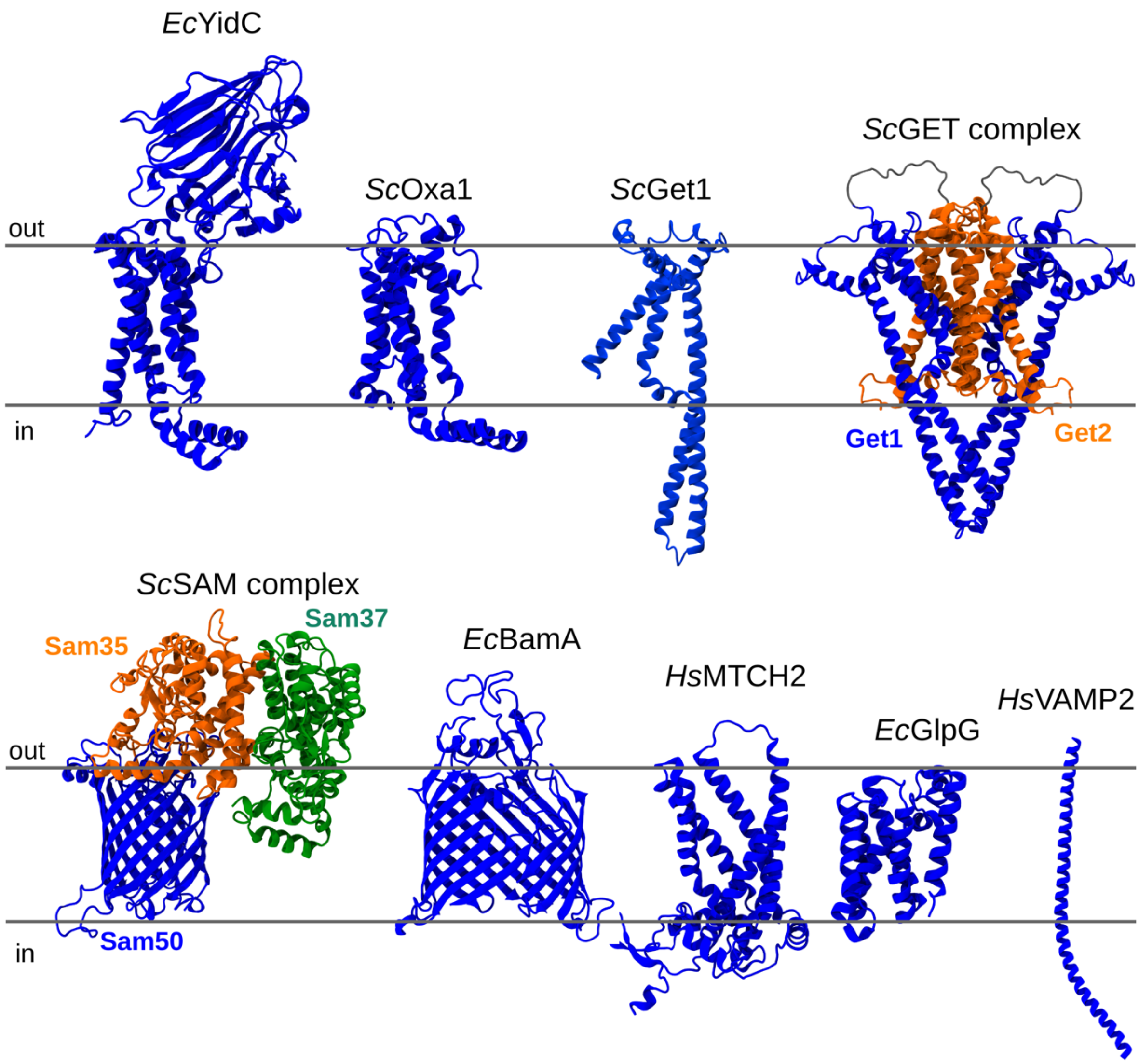

Figure S1. 3D structure of experimentally tested proteins.

bioRxiv preprint doi: https://doi.org/10.1101/2023.09.01.555937; this version posted September 1, 2023. The copyright holder for this preprint (which was not certified by peer review) is the author/funder, who has granted bioRxiv a license to display the preprint in perpetuity. It is made available under aCC-BY 4.0 International license.

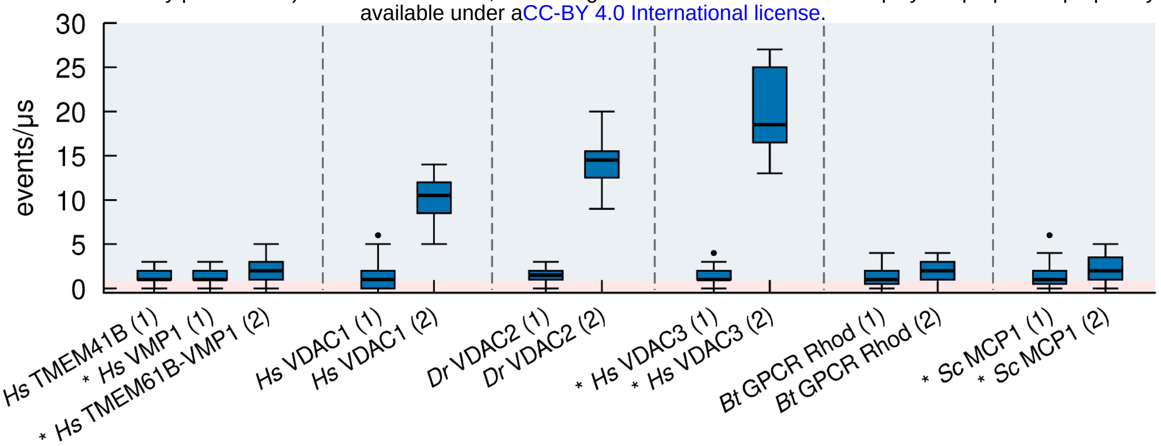

Figure S2. Oligomerization influences lipid scrambling. Comparison of lipid scrambling activity between monomeric and dimeric forms of selected scramblases. The blue shaded region represents the behavior for a scrambling-positive protein and the red area for a scrambling-negative protein. AlphaFold structures are denoted by the * symbol, oligomerization state is in parenthesis.

bioRxiv preprint doi: https://doi.org/10.1101/2023.09.01.555937; this version posted September 1, 2023. The copyright holder for this preprint (which was not certified by peer review) is the author/funder, who has granted bioRxiv a license to display the preprint in perpetuity. It is made available under aCC-BY 4.0 International license.

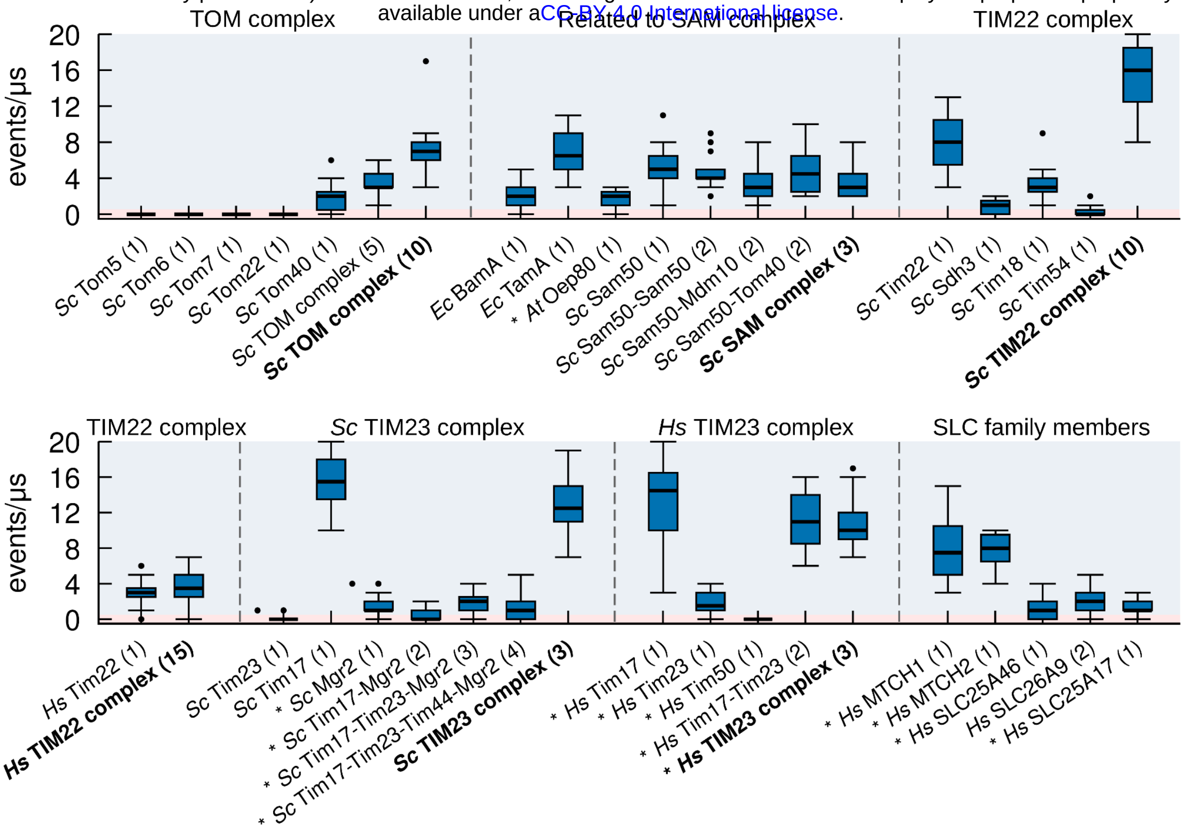

Figure S3. Extended version of insertase complexes in the mitochondria. Lipid scrambling activity of individual components and selected complexes for the major mitochondrial insertases investigated. The blue shaded region represents the behavior for a scrambling-positive protein and the red area for a scrambling-negative protein. AF structures are denoted by the ***** symbol, oligomerization state is in parenthesis.

bioRxiv preprint doi: https://doi.org/10.1101/2023.09.01.555937; this version posted September 1, 2023. The copyright holder for this preprint (which was not certified by peer review) is the author/funder, who has granted bioRxiv a license to display the preprint in perpetuity. It is made available under aCC-BY 4.0 International license.

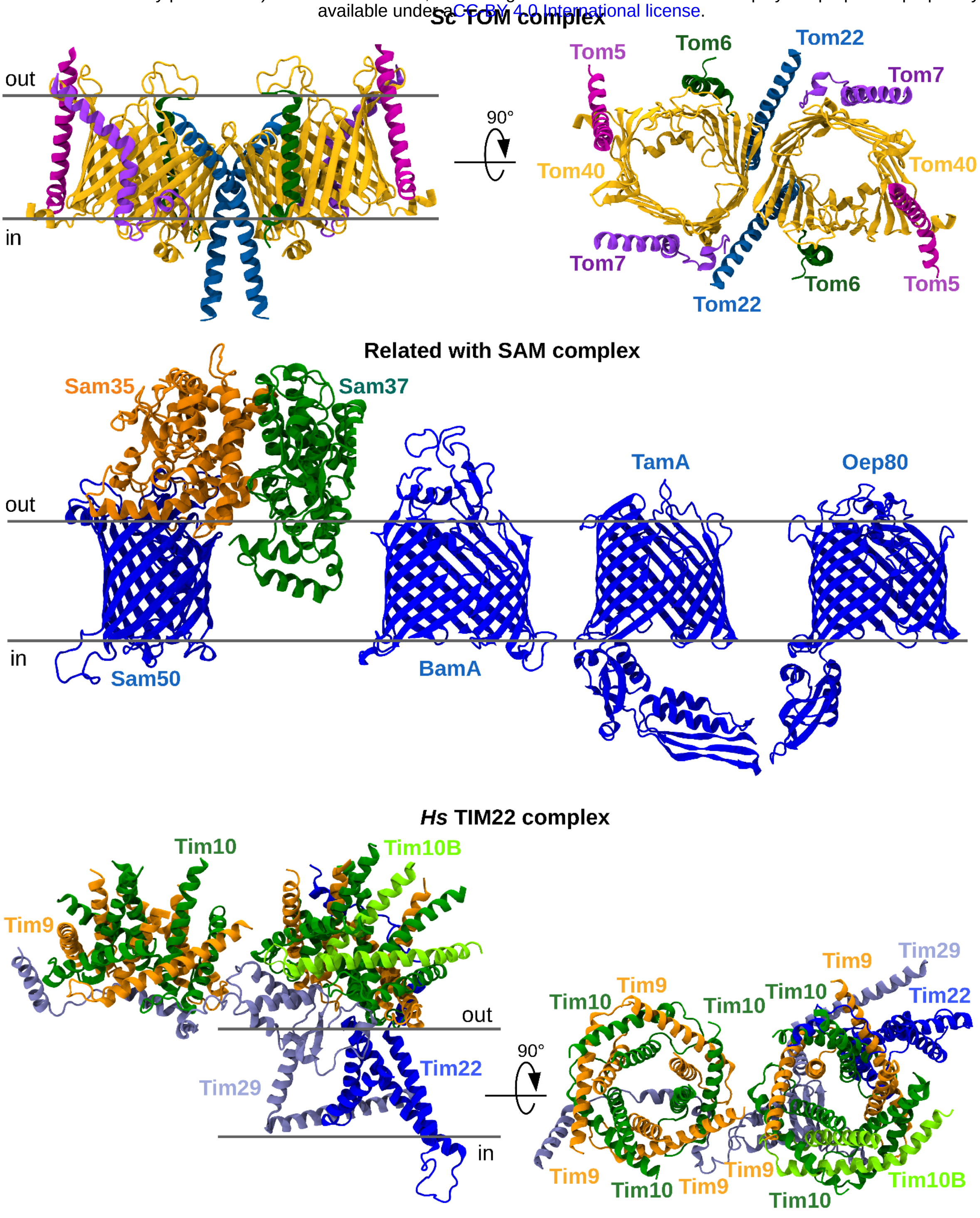

Figure S4. 3D structures of mitochondrial complexes TOM, SAM and *Hs*TIM22.

bioRxiv preprint doi: https://doi.org/10.1101/2023.09.01.555937; this version posted September 1, 2023. The copyright holder for this preprint (which was not certified by peer review) is the author/funder, who has granted bioRxiv a license to display the preprint in perpetuity. It is made available under aCC-BY 4.0 International license.

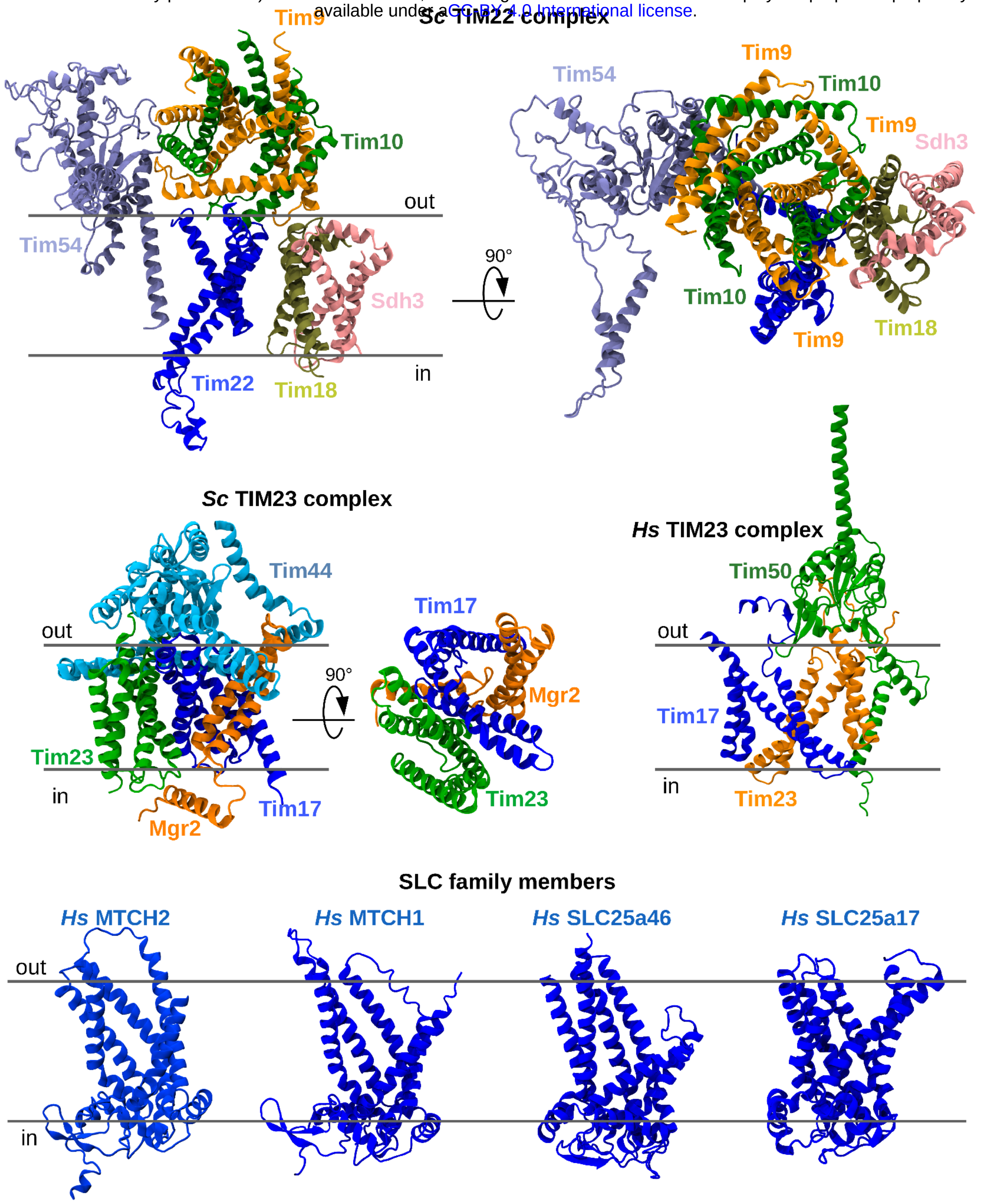

Figure S5. 3D structures of mitochondrial complexes *Sc*TIM22, *Sc*TIM23, *Hs*TIM23 and SLC proteins MTCH2, MTCH1, SLC25a46 and SLC25a17.

bioRxiv preprint doi: https://doi.org/10.1101/2023.09.01.555937; this version posted September 1, 2023. The copyright holder for this preprint (which was not certified by peer review) is the author/funder, who has granted bioRxiv a license to display the preprint in perpetuity. It is made available under aCC-BY 4.0 International license.

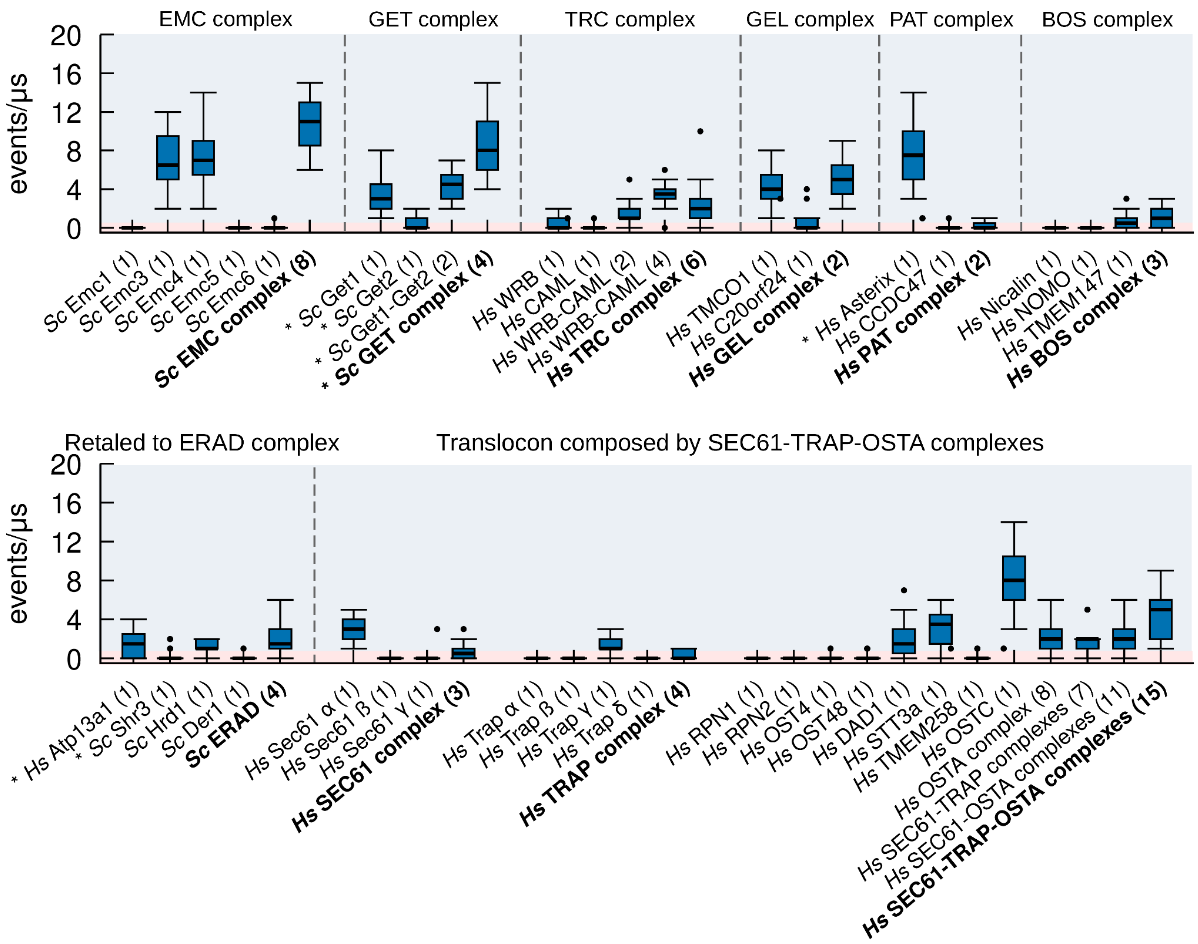

Figure S6. Extended version of insertase complexes in the endoplasmic reticulum. Lipid scrambling activity of individual components and selected complexes for the major ER insertases investigated. The blue shaded region represents the behavior for a scrambling-positive protein and the red area for a scrambling-negative protein. AlphaFold structures are denoted by the * symbol, oligomerization state is in parenthesis.

bioRxiv preprint doi: https://doi.org/10.1101/2023.09.01.555937; this version posted September 1, 2023. The copyright holder for this preprint (which was not certified by peer review) is the author/funder, who has granted bioRxiv a license to display the preprint in perpetuity. It is made available under aCC-BY 4.0 International license.

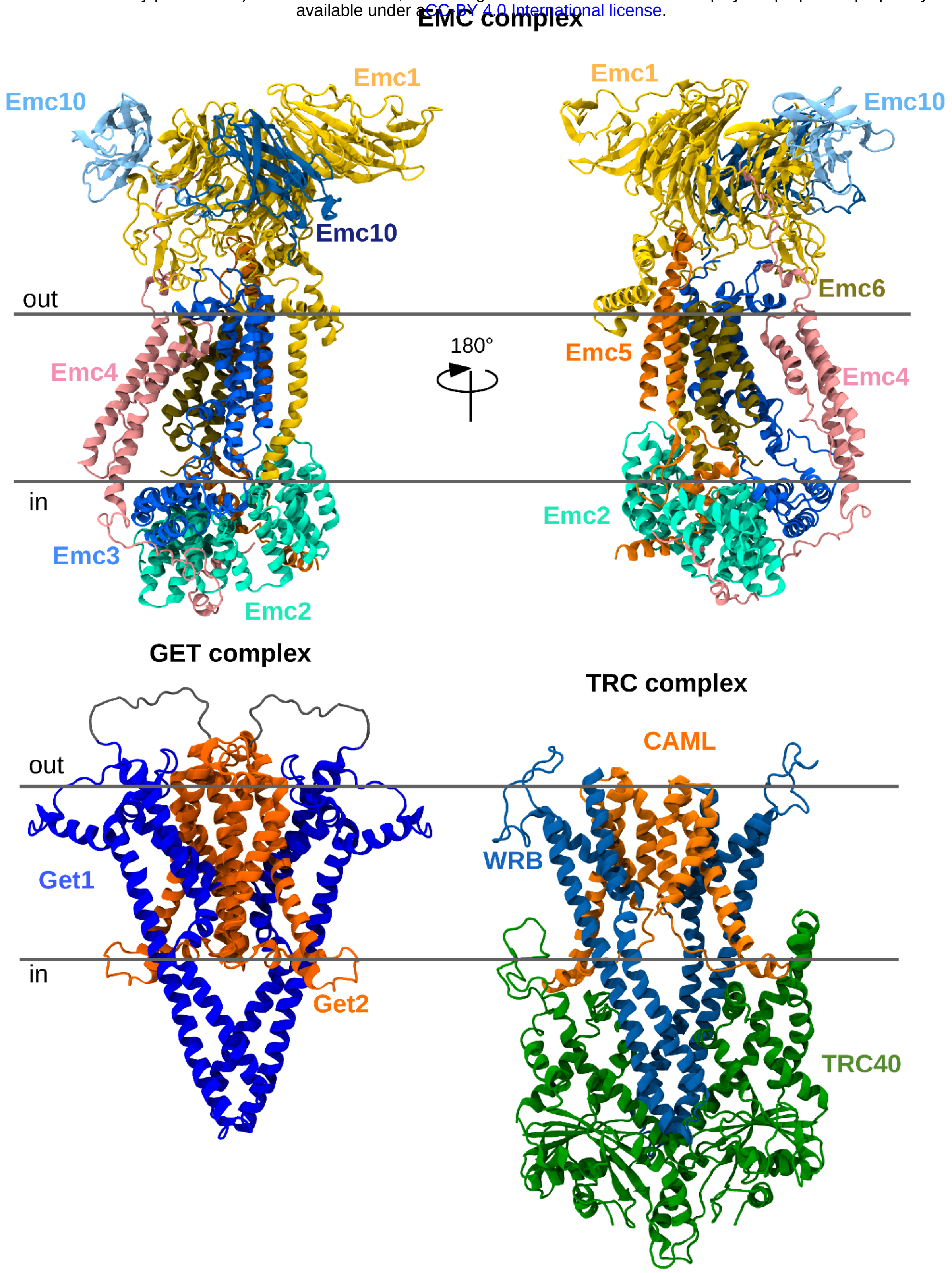

Figure S7. 3D structures of ER complexes EMC, GET and TRC.

bioRxiv preprint doi: https://doi.org/10.1101/2023.09.01.555937; this version posted September 1, 2023. The copyright holder for this preprint (which was not certified by peer review) is the author/funder, who has granted bioRxiv a license to display the preprint in perpetuity. It is made available under aCC-BY 4.0 International license.

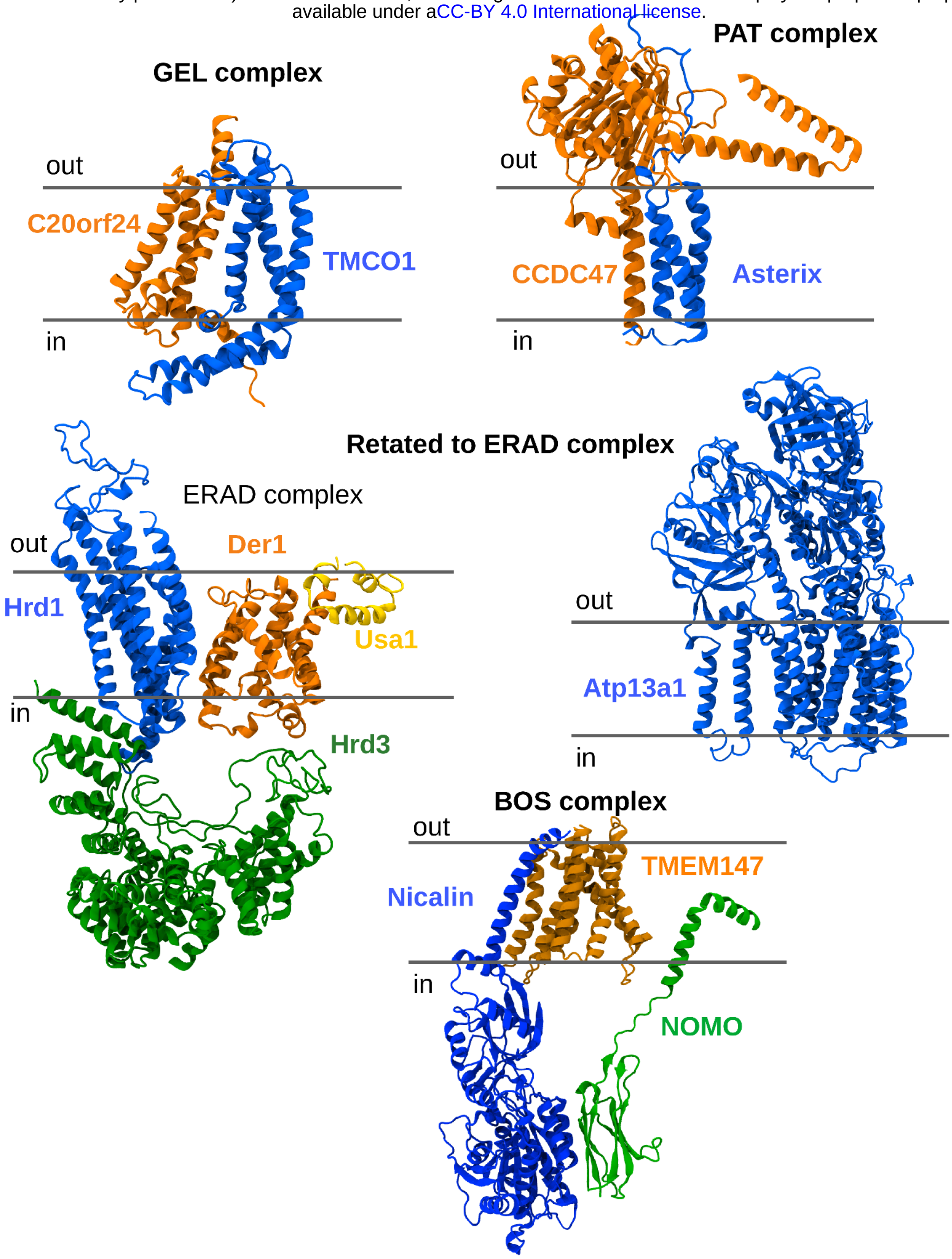

Figure S8. 3D structures of ER complexes GEL, PAT, ERAD, Atp13a1 and BOS complex.

bioRxiv preprint doi: https://doi.org/10.1101/2023.09.01.555937; this version posted September 1, 2023. The copyright holder for this preprint (which was not certified by peer review) is the author/funder, who has granted bioRxiv a license to display the preprint in perpetuity. It is made available under aCC-BY 4.0 International license.

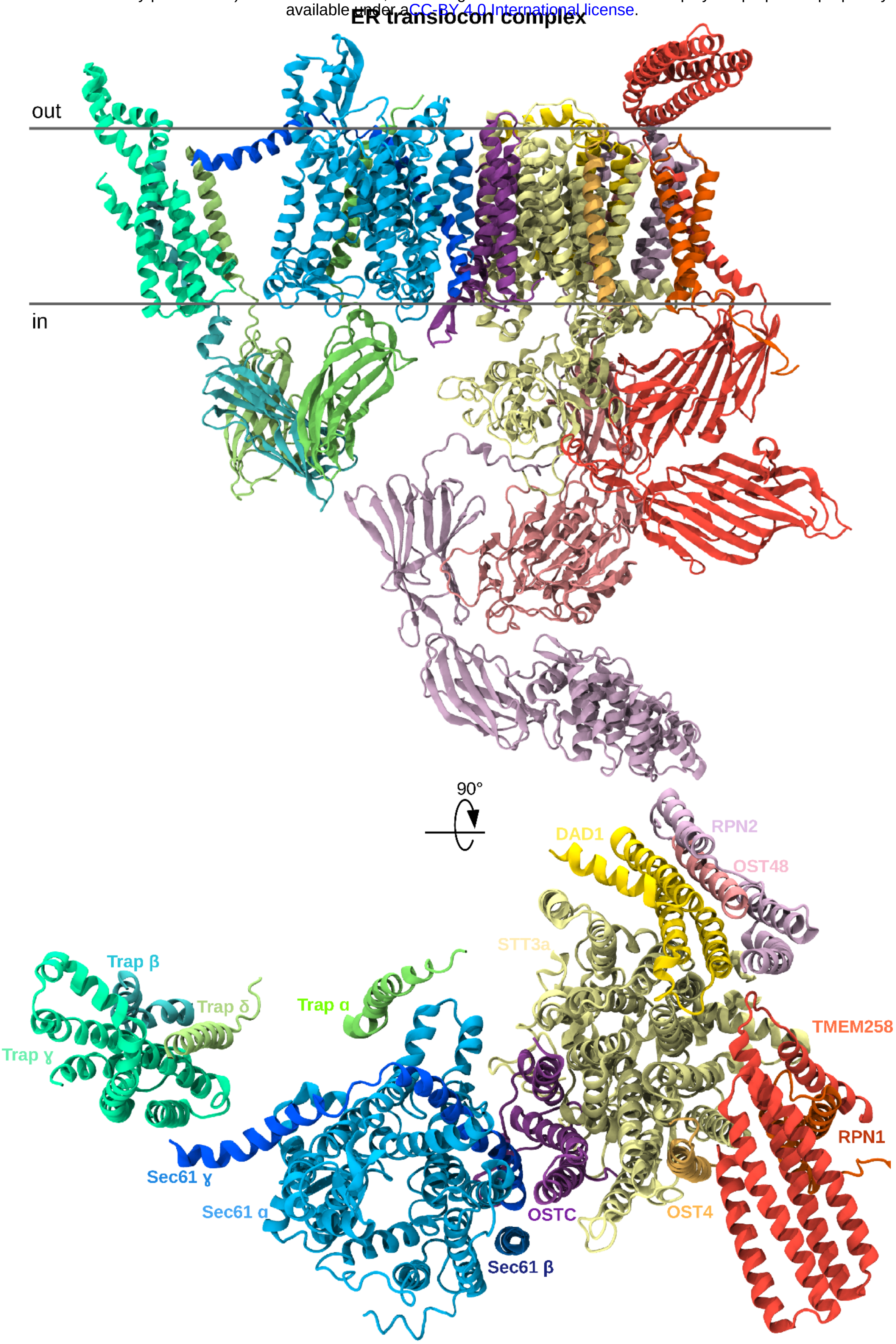

Figure S9. 3D structure of *Hs*SEC61-TRAP-OSTA complex.

bioRxiv preprint doi: https://doi.org/10.1101/2023.09.01.555937; this version posted September 1, 2023. The copyright holder for this preprint (which was not certified by peer review) is the author/funder, who has granted bioRxiv a license to display the preprint in perpetuity. It is made available under aCC-BY 4.0 International license.

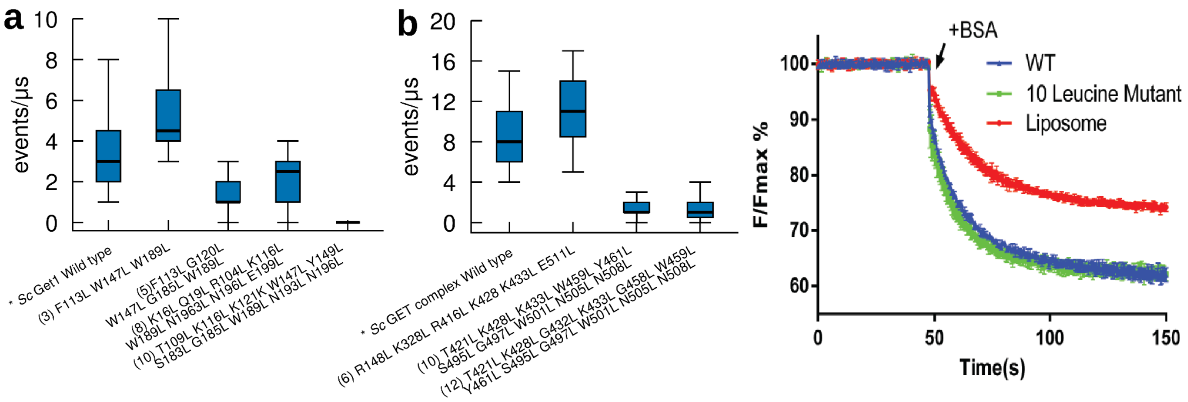

Figure S10. Mutational analysis of lipid scrambling in Get1 and GET complex. a. Scrambling activity calculations from CG-MD simulations of the wild type and various mutants of the *Sc*Get1 subunit. 10 mutations (10L) are required to completely ablate lipid scrambling activity in *Sc*Get1 **b.** Left. Scrambling activity calculations from CG-MD simulations of the wild type and various mutants of the *Sc*GET complex. Neither the same 10L mutations on Get1 and a new 12L mutations were able to ablate lipid scrambling activity in silico in the *Sc*GET complex. Right. Lipid scrambling activity of wt and L10 mutant GET. The scramblase assay for Get2-1sc mutant was carried out with FluoroMax+ spectrofluorometer (HORIBA). For each reaction, 50uL of the proteoliposomes were added to 1950uL of the reconstitution buffer. The sample was vigorously stirred and measured for fluorescence at 460/538 nm for 50-70 seconds to establish a stable baseline. 50uL of 1.5mg/mL fatty acid-free BSA was added, and fluorescence data were collected for another 200s. Both the WT complex and the mutant version with 10 mutations in the translocation channel (T421L/K428L/K433L/W459L/Y461L/S495L/G497L/W501L/N505L/N508L in Get1) scramble NBD-PC. **a-b**. The total number of mutations is in parentheses.

bioRxiv preprint doi: https://doi.org/10.1101/2023.09.01.555937; this version posted September 1, 2023. The copyright holder for this preprint (which was not certified by peer review) is the author/funder, who has granted bioRxiv a license to display the preprint in perpetuity. It is made available under aCC-BY 4.0 International license.

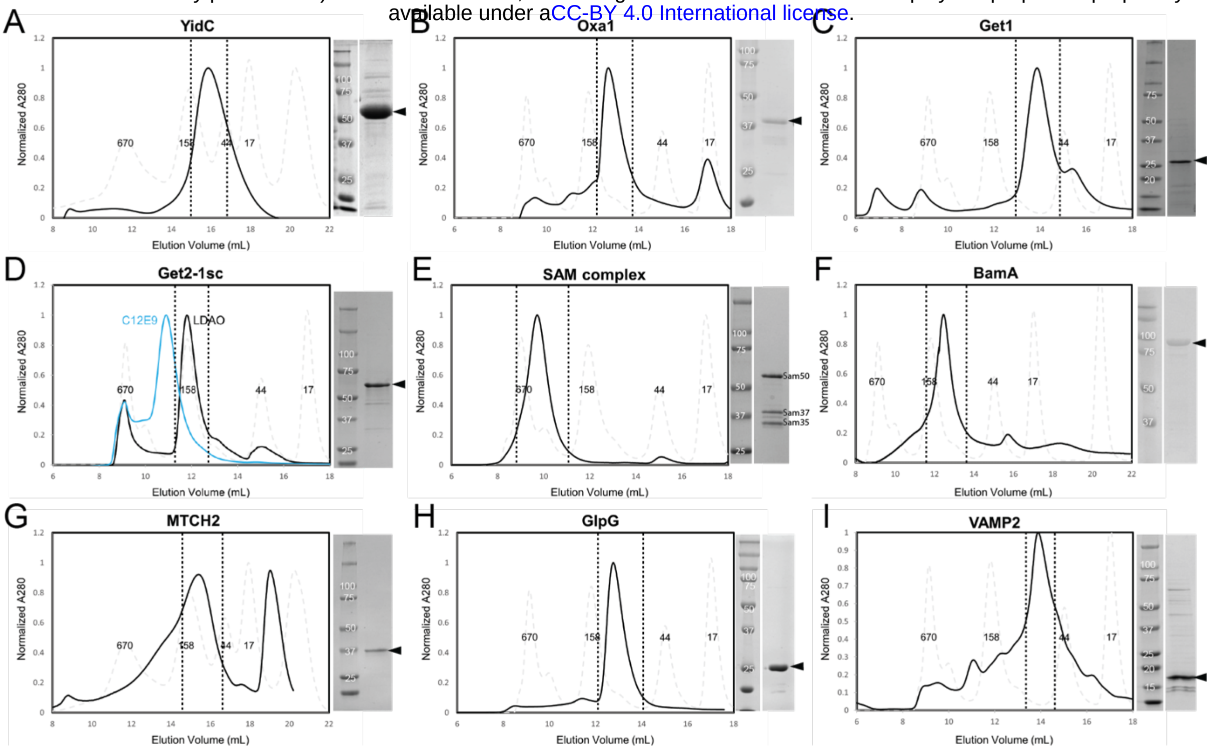

Figure S11. Size-exclusion chromatography analyses of proteins used in reconstitutions. Proteins for in vitro reconstitution were purified by size exclusion chromatography. Traces are shown (black) along with traces of the molecular weight standards (dotted grey); fractions used for reconstitutions are indicated (dotted black lines). Note that the elution volume of each protein reflects its size including detergent micelle. SDS-PAGE analysis of the purified protein is to the right of each chromatographic trace.
